# Snakebite Advice and Counseling From Artificial Intelligence: An Acute Venomous Snakebite Consultation With ChatGPT

**DOI:** 10.7759/cureus.40351

**Published:** 2023-06-13

**Authors:** Ibraheem Altamimi, Abdullah Altamimi, Abdullah S Alhumimidi, Abdulaziz Altamimi, Mohamad-Hani Temsah

**Affiliations:** 1 College of Medicine, King Saud University, Riyadh, SAU; 2 Pediatric Emergency and Toxicology Department, King Fahd Medical City, Riyadh, SAU; 3 College of Medicine, King Saud Bin Abdulaziz University for Health Sciences, Riyadh, SAU; 4 Pediatric Intensive Care Unit, Pediatric Department, King Saud University Medical City, Riyadh, SAU

**Keywords:** ai and machine learning, medical artificial intelligence, artificial intelligence and education, ai chatbot, emergency medicine, toxicology and envenomation, acute venomous snakebite, artificial intelligence in healthcare, chatgpt, snake-bite

## Abstract

Background: Snakebites, particularly from venomous species, present a significant global public health challenge. Access to accurate and timely information regarding snakebite prevention, recognition, and management is crucial for minimizing morbidity and mortality. Artificial intelligence (AI) language models, such as ChatGPT (Chat Generative Pre-trained Transformer), have the potential to revolutionize the dissemination of medical information and improve patient education and satisfaction.

Methods: This study aimed to explore the utility of ChatGPT, an advanced language model, in simulating acute venomous snakebite consultations. Nine hypothetical questions based on comprehensive snakebite management guidelines were posed to ChatGPT, and the responses were evaluated by clinical toxicologists and emergency medicine physicians.

Results: ChatGPT provided accurate and informative responses related to the immediate management of snakebites, the urgency of seeking medical attention, symptoms, and health issues following venomous snakebites, the role of antivenom, misconceptions about snakebites, recovery, pain management, and prevention strategies. The model highlighted the importance of seeking professional medical care and adhering to healthcare practitioners' advice. However, some limitations were identified, including outdated knowledge, lack of personalization, and inability to consider regional variations and individual characteristics.

Conclusion: ChatGPT demonstrated proficiency in generating intelligible and well-informed responses related to venomous snakebites. It offers accessible and real-time advice, making it a valuable resource for preliminary information, education, and triage support in remote or underserved areas. While acknowledging its limitations, such as the need for up-to-date information and personalized advice, ChatGPT can serve as a supplementary source of information to complement professional medical consultation and enhance patient education. Future research should focus on addressing the identified limitations and establishing region-specific guidelines for snakebite management.

## Introduction

Snakebite incidents, particularly those involving venomous species, pose a significant public health challenge worldwide [1]. Venomous snakebites can result in severe local and systemic complications, including pain, swelling, tissue damage, coagulopathy, and even death [2]. Timely recognition and management of venomous snakebites are crucial to minimize morbidity and mortality. Patients and caregivers often have concerns about the identification, symptoms, prevention, and treatment of snakebites, which they may feel hesitant to ask about or may not even realize the need to inquire about [3].

Artificial intelligence (AI) language-generated tools, such as ChatGPT (Chat Generative Pre-trained Transformer), present a remarkable opportunity to revolutionize the dissemination of medical information. ChatGPT is an advanced language model with the ability to generate human-like text, attracting interest for its potential to assist researchers in writing scientific papers and conducting literature reviews [4]. By utilizing extensive text data from various sources on the internet, ChatGPT can provide logical, coherent, and accurate responses to a wide range of questions related to medical topics [5].

Despite the rapid advancements in AI, there is a limited understanding of its potential benefits for addressing public inquiries, particularly in the context of acute medical conditions like venomous snakebites [6]. To explore this potential, the authors conducted a simulated acute venomous snakebite consultation using ChatGPT to generate answers to questions commonly posed by patients and caregivers, subsequently evaluating the responses. The authors argue that the integration of AI and language models, such as ChatGPT, in medical consultations has significant potential for improving patient education and satisfaction. As AI and machine learning technologies continue to progress, they may open new avenues for innovative approaches to enhance patient outcomes in scenarios involving venomous snakebites and other acute medical conditions.

## Materials and methods

In this study, we aimed to investigate the potential of AI language models to serve as clinical assistants in the context of venomous snakebites. For this purpose, we employed ChatGPT, one of the largest language models currently accessible to the public, and evaluated its capacity, effectiveness, and accuracy in providing information related to the prevention, recognition, and management of acute venomous snakebite incidents.

Study design

We asked ChatGPT nine hypothetical questions simulating a doctor-patient and doctor-doctor consultation for venomous snakebites. The questions were based on comprehensive snakebite management information guidelines derived from reputable sources such as the World Health Organization, Centers for Disease Control and Prevention, and the clinical literature [7-9]. The scenario objective was to cover a broad cross-section of information that patients or caregivers might want to know regarding venomous snakebites. The responses provided by ChatGPT were assessed for accuracy, informativeness, and accessibility by clinical toxicologists and emergency medicine physicians with extensive experience in managing acute snakebite cases.

By rigorously evaluating these responses according to the criteria, we sought to establish the utility of AI-generated language models, such as ChatGPT, in simulating doctor-patient consultations for venomous snakebites and other acute medical conditions.

Inclusion and exclusion criteria

ChatGPT employs a probabilistic algorithm, utilizing random sampling to generate a diverse array of responses, which may lead to different answers to identical questions. For the purpose of this study, we selected ChatGPT 3.5, a version that is both widely available and freely accessible to the public. ChatGPT 4 was excluded from consideration due to its limited availability and the costs associated with its use [10].

We documented the initial response provided by ChatGPT 3.5 for each question and refrained from allowing any subsequent clarifications or corrections. Meticulous attention was given to ensuring that all questions were devoid of grammatical or syntactical errors. Each question was input on the same day using a single ChatGPT 3.5 account, which was maintained by one of the authors.

Alternative chatbots were not considered for this study, as they demonstrated inferior capabilities when compared to ChatGPT [11]. The primary objective was to assess the performance of ChatGPT 3.5 as a representative of advanced AI-generated language models within the specific context of consultations related to venomous snakebites.

## Results

**Figure 1:**
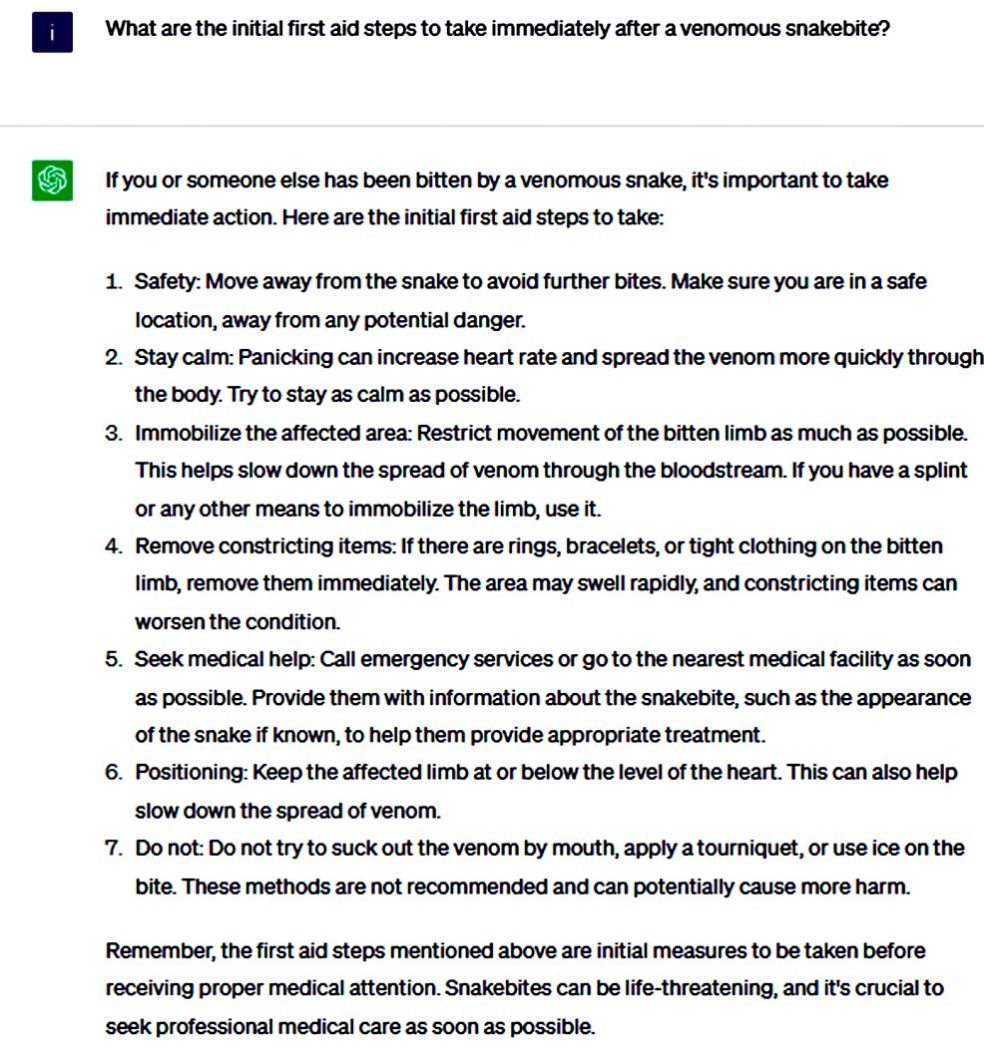
What are the initial first aid steps to take immediately after a venomous snakebite?

At the commencement of the consultation, we inquired about ChatGPT's recommendations for the immediate management of a patient who has been bitten by a snake and the appropriate actions to take under such circumstances (Figure 1).

**Figure 2:**
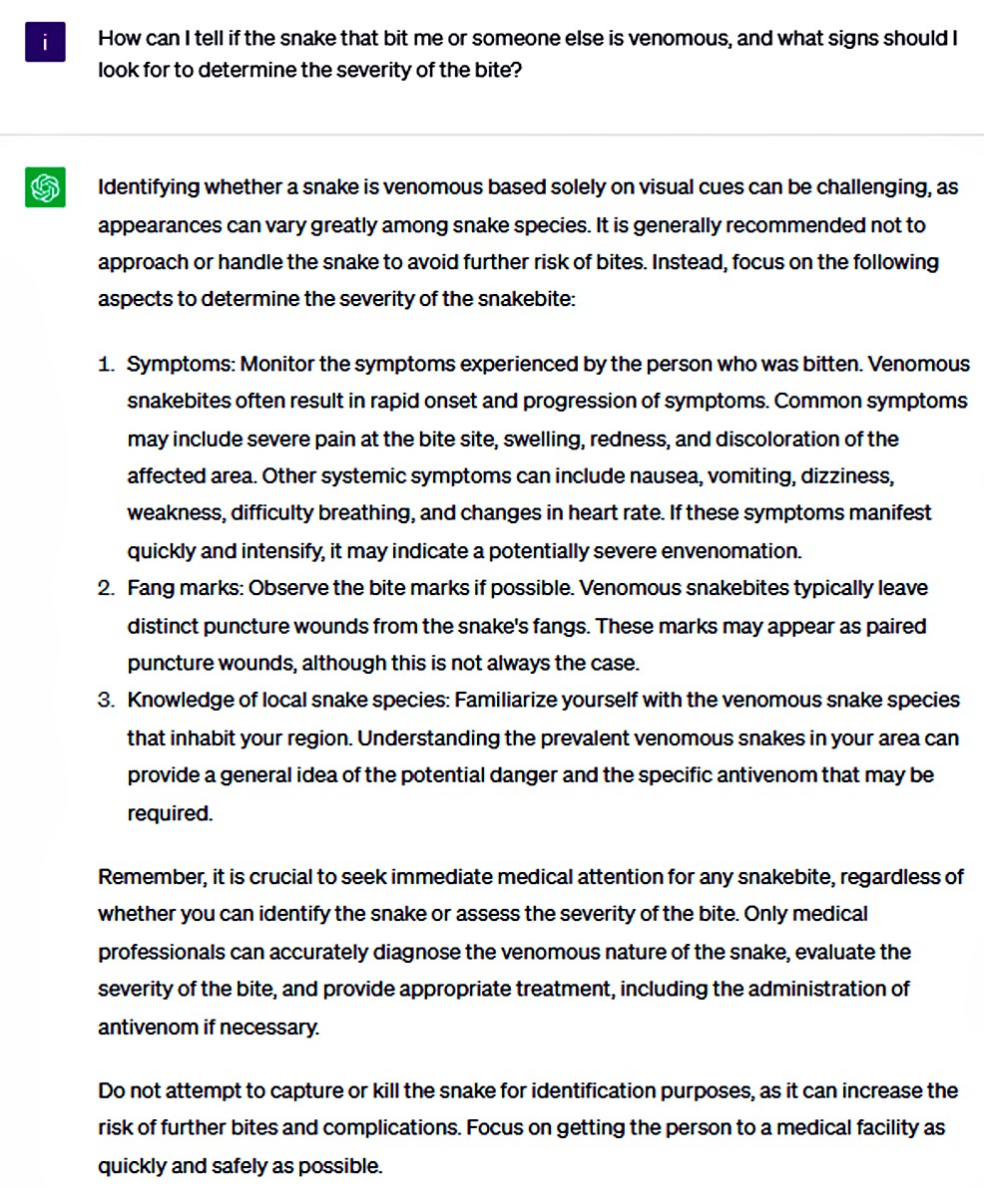
How can I tell if the snake that bit me or someone else is venomous, and what signs should look for to determine the severity of the bite?

Followed by a question regarding the urgency of seeking medical attention after a snakebite and whether the advised timeline differs depending on the snake's venomous status (Figure 2).

ChatGPT underscored the vital importance of promptly seeking professional medical care following a snakebite, as the preliminary steps taken at the scene may only provide limited relief and are not a substitute for comprehensive medical evaluation and treatment. Medical professionals are equipped with the knowledge and resources to accurately assess the severity of the bite, administer appropriate interventions such as antivenom if required, and monitor the patient for any potential complications arising from the snakebite, ensuring the best possible outcome. ChatGPT provided a series of evidence-based initial measures and actions to be avoided following snakebites, offering accurate and reliable guidance that aligns with the recommendations from authoritative sources, such as [7]. Conversely, ChatGPT did not prioritize the most critical measures, presenting them as an unordered list rather than emphasizing their relative importance. Consequently, patients may follow certain recommendations while neglecting others. It is essential to highlight the level of significance for each measure instead of presenting them as a mere checklist, ensuring that patients understand and adhere to the most vital steps in managing a snakebite.

**Figure 3:**
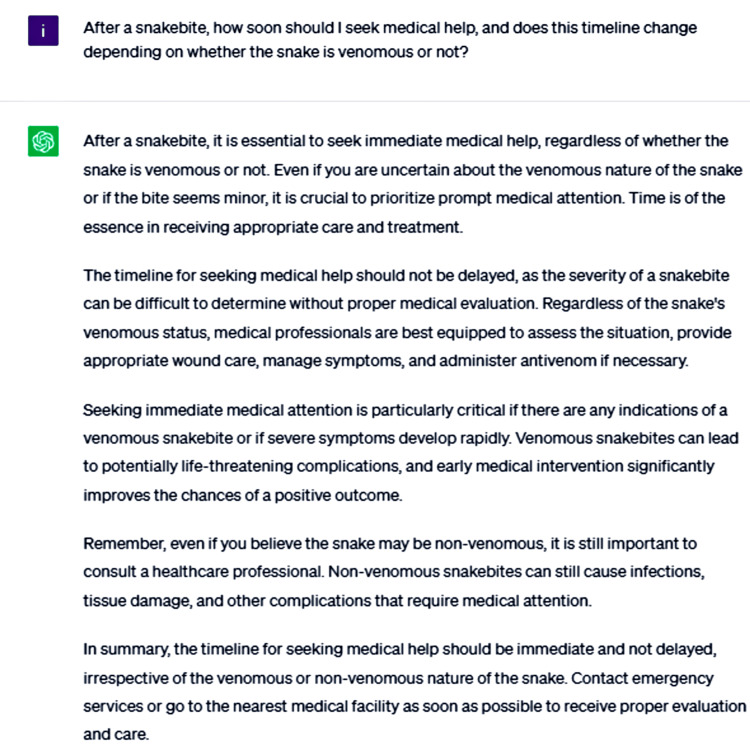
After a snakebite, how soon should I seek medical help, and does this timeline change depending on whether the snake is venomous or not?

ChatGPT was prompted to provide more detailed information through three additional questions. The first query asked about the urgency of seeking medical help after a snakebite and whether this timeline changes depending on the snake's venomous status (Figure 3).

**Figure 4:**
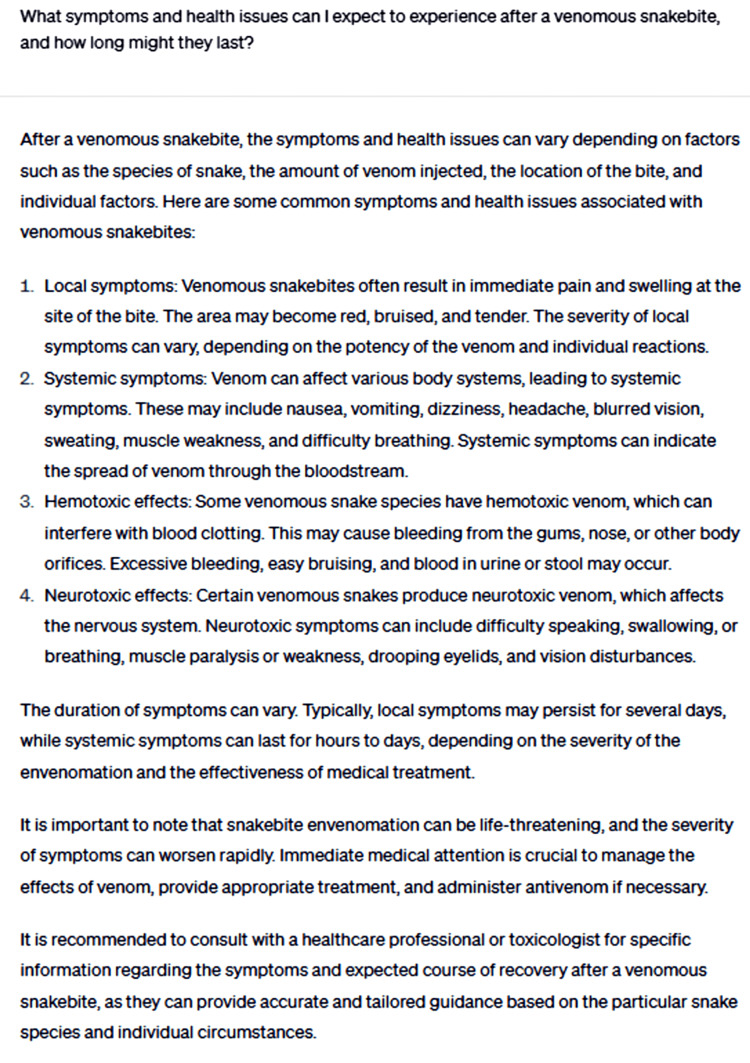
What symptoms and health issues can I expect to experience after a venomous snakebite, and how long might they last?

The second question inquired about the symptoms and health issues one can expect to experience after a venomous snakebite and the duration of these symptoms (Figure 4).

**Figure 5:**
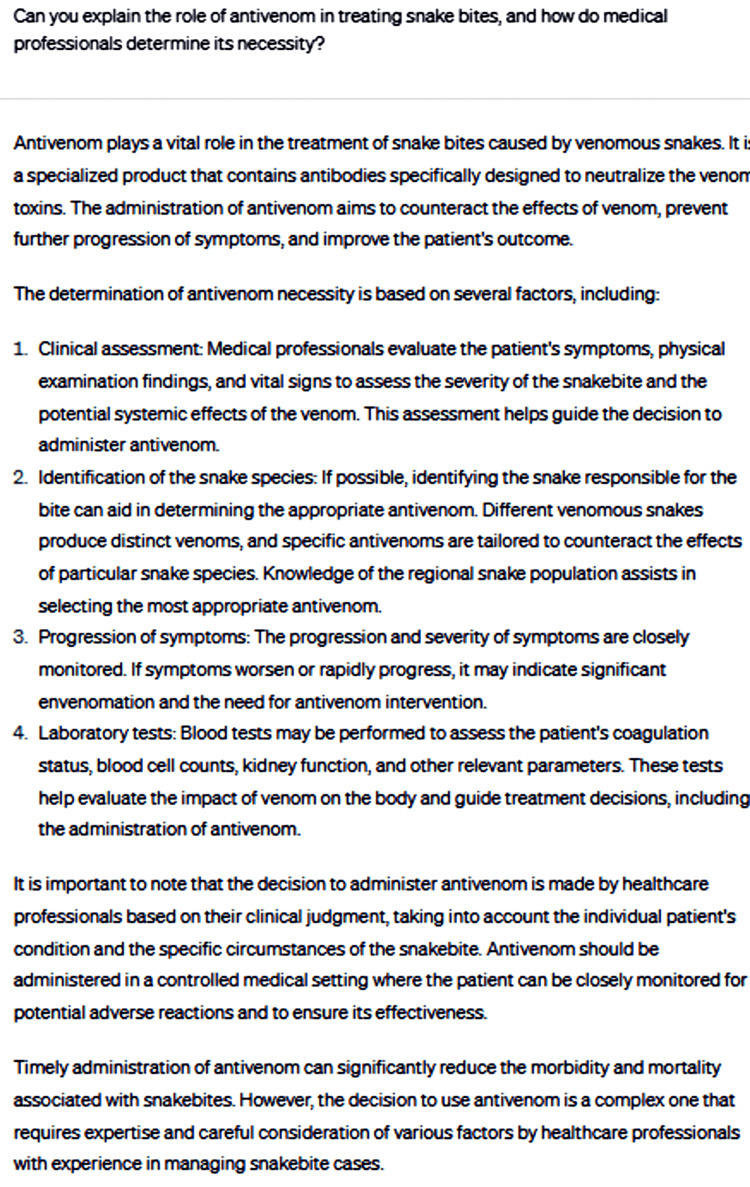
Can you explain the role of antivenom in treating snakebites, and how do medical professionals determine its necessity?

Lastly, ChatGPT was asked to explain the role of antivenom in treating snakebites and how medical professionals determine its necessity for a specific case (Figure 5).

It is important to note that ChatGPT serves as a tool to assist patients and clinicians with information and guidance; however, it cannot be relied upon to manage patients independently. We strongly emphasize the need for patients to seek medical attention without delay in cases of snakebites or other emergencies. One of the strengths of large language models like ChatGPT is the ability to recognize their limitations, ensuring that users understand the necessity of professional medical intervention in critical situations [12].

The advice provided emphasizes the crucial role of antivenom in treating venomous snakebites and highlights the significance of clinical assessment, snake identification, symptom progression, and laboratory tests in guiding treatment decisions. It also underlines the importance of timely administration of antivenom to reduce morbidity and mortality associated with snakebites. Additionally, the advice acknowledges the need for expertise and careful consideration in making the decision to administer antivenom, reinforcing the importance of seeking professional medical care in such situations. The advice, being general in nature, may not cover specific regional variations in snake species, venom characteristics, and availability of antivenom. This generality, along with the emphasis on the potential severity of snakebites and the need for antivenom, might cause anxiety for patients, particularly if antivenom is not readily accessible or if the snake species involved is unknown. Furthermore, patients might misinterpret the advice and attempt to self-diagnose or manage their treatment, potentially leading to delays in seeking professional help or receiving inadequate care.

**Figure 6:**
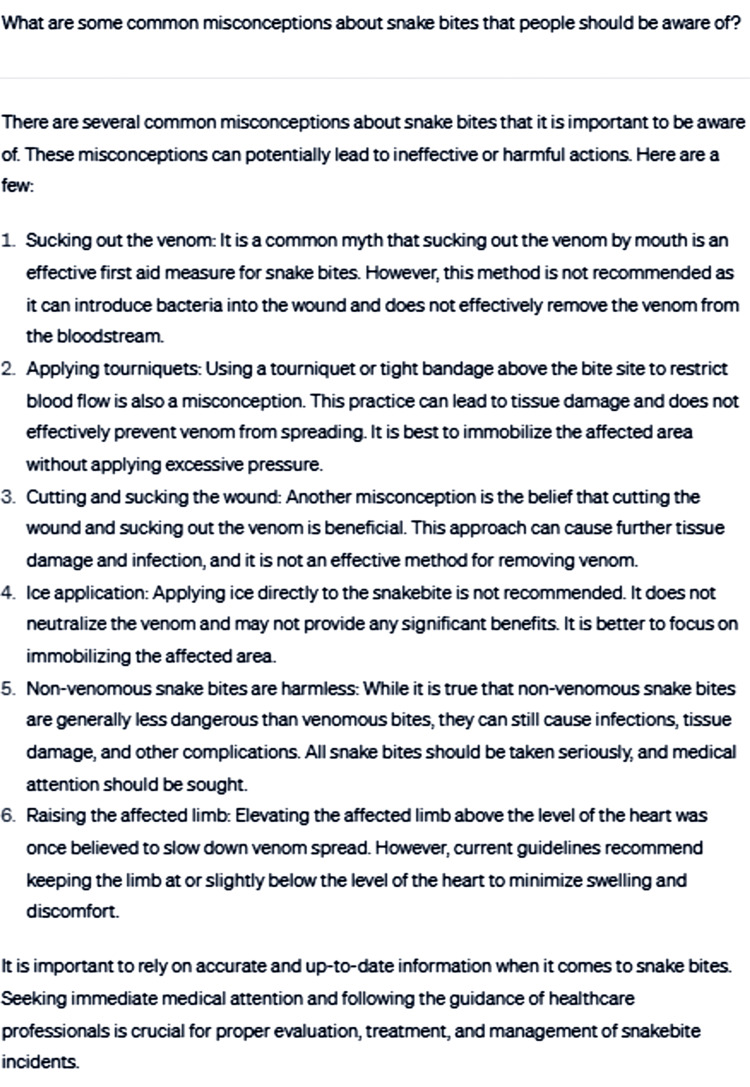
What are some common misconceptions about snakebites that people should be aware of?

The remainder of the consultation encompassed various aspects related to venomous snakebites, including recovery, prevention strategies, and addressing misconceptions. Initially, ChatGPT was asked, "What are some common misconceptions about snakebites that people should be aware of?" in order to provide clarity and dispel potential misinformation (Figure 6).

**Figure 7:**
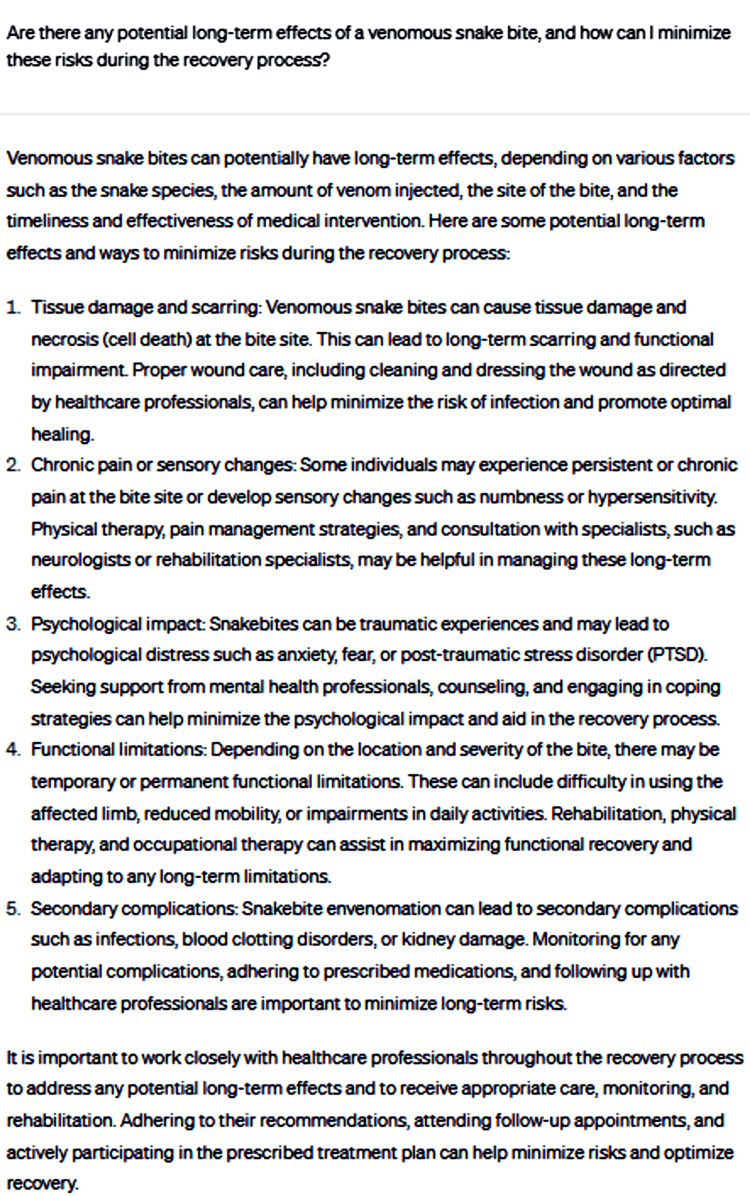
Are there any potential long-term effects of a venomous snakebite, and how can I minimize these risks during the recovery process?

Subsequently, the discussion focused on the potential long-term effects of venomous snakebites and methods to minimize risks during the recovery process (Figure 7).

**Figure 8:**
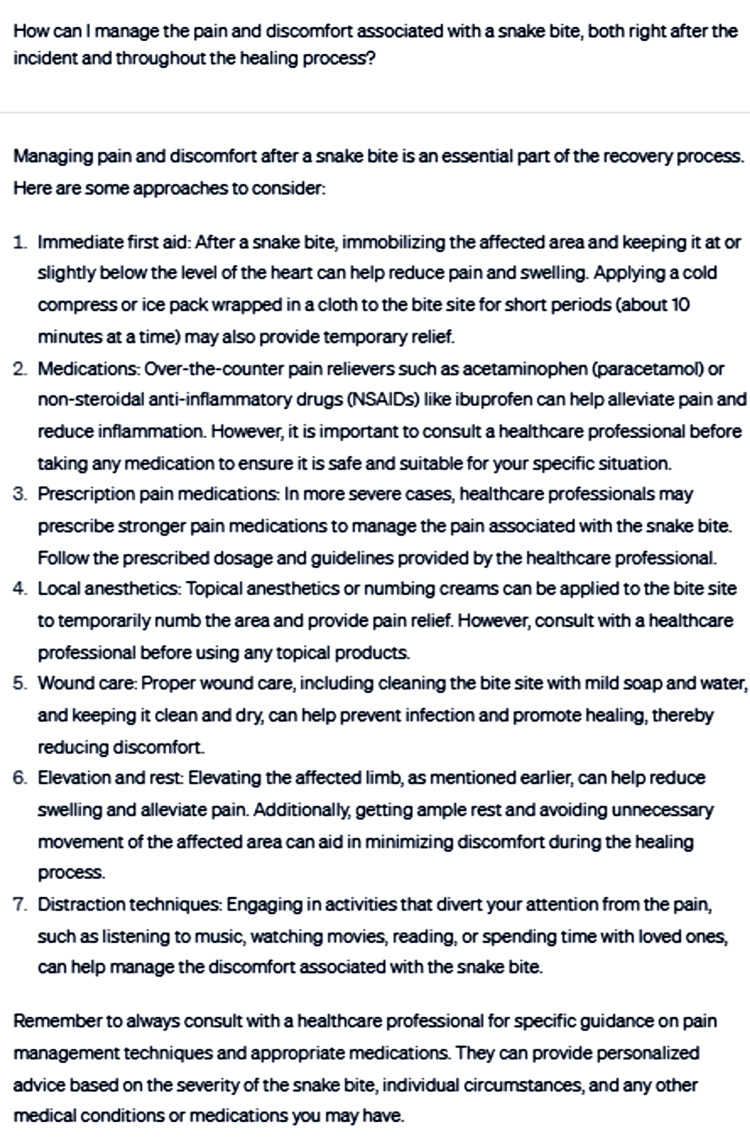
How can I manage the pain and discomfort associated with a snakebite, both right after the incident and throughout the healing process?

Additionally, the conversation explored managing pain and discomfort associated with snakebites, both immediately following the incident and throughout the healing process (Figure 8).

**Figure 9:**
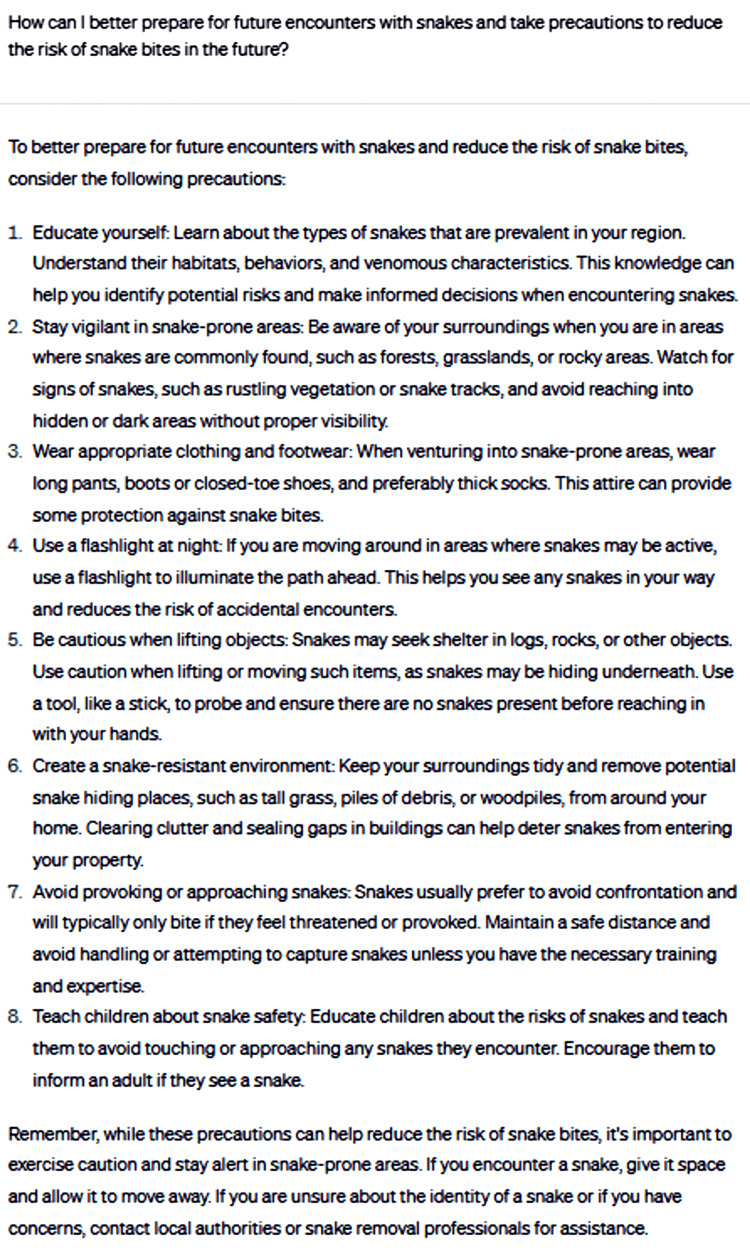
How can I better prepare for future encounters with snakes and take precautions to reduce the risks of snakebites in the future?

Lastly, the consultation delved into effective preparation for future encounters with snakes and adopting precautions to mitigate the risk of snakebites in the future (Figure 9).

The response effectively addresses widespread myths and misconceptions associated with snakebites, helping to reduce misinformation. It offers evidence-based guidance based on current guidelines and practices, ensuring the advice is reliable and accurate [7,9,13]. Additionally, the response emphasizes the importance of seeking immediate medical attention and following the guidance of healthcare professionals, which is crucial for the proper evaluation, treatment, and management of snakebites. However, there are some limitations to the response, such as limited depth on toxicology, as it does not delve into the specific toxicological aspects of venomous snakebites, like the type of venom, how it affects the body or the administration of antivenom. Furthermore, the reply does not address the variation in venom toxicity and severity of symptoms between different snake species, which could be important information depending on the region or type of snake involved.

## Discussion

This exploratory research delved into ChatGPT's capabilities to understand and generate responses in natural language focused on the healthcare domain, specifically targeting consultations and recommendations related to acute venomous snakebites. The AI model exhibited proficiency in creating intelligible, well-informed responses that were easily comprehensible and grounded in factual information [14]. While recognizing its own constraints, ChatGPT persistently underscored the necessity of seeking immediate medical care and following the advice of healthcare practitioners in handling snakebite incidents [12].

ChatGPT offers numerous benefits in the context of providing medical advice for venomous snakebites. Its accessibility through a variety of digital platforms allows individuals in remote or underserved areas to acquire preliminary information and advice concerning snakebites. The AI model delivers real-time responses to inquiries, granting users prompt guidance in snakebite situations. With its natural language generation, ChatGPT ensures that its advice is easily comprehensible for users without specialized medical knowledge. Owing to its diverse training sources, ChatGPT can offer recommendations on multiple aspects of snakebite management, such as first aid, potential complications, and recovery. The model also presents consistent information and advice based on its training data, mitigating the risk of human error or variability in responses. In cases where healthcare resources are scarce, ChatGPT can provide triage support by offering general guidance on snakebite severity and recommended actions, potentially minimizing unnecessary hospital visits. Furthermore, ChatGPT functions as an educational resource for the general public and healthcare professionals, delivering information on snakebite prevention, identification, and management.

However, several limitations are associated with ChatGPT's provision of medical advice for venomous snakebites. Its knowledge, constrained by training data and a knowledge cutoff, lacks real-time updates, which may result in out-of-date or incomplete advice [15]. ChatGPT is incapable of delivering personalized advice that considers an individual's medical history, allergies, or other unique factors, thereby potentially restricting the utility of its recommendations. The AI model might not fully comprehend context, leading to potential misunderstandings or unsuitable advice, such as failing to recognize regional variations in snake species and venom toxicity or neglecting specific patient information. ChatGPT's limited clinical judgment compared to healthcare professionals may result in overlooking the seriousness of a situation or offering inadequate guidance [4]. The model also cannot evaluate visual information, such as images of the snake or the bite, which impedes its capacity to provide accurate advice. AI's inherent lack of empathy and compassion renders its advice impersonal and less emotionally supportive [16]. Moreover, liability and ethical concerns emerge when relying on AI for medical advice, as misinterpretations or inaccuracies might lead to detrimental outcomes, raising questions about responsibility and accountability [17].

Limitations and future directions

In our study, we identified several limitations and potential future directions for further exploration and research. Our investigation primarily focused on the patient perspective, omitting the physician's point of view and simulated cases, which could offer additional insights. The assessments relied on expert opinions instead of a formal grading rubric or qualitative analysis, potentially introducing subjectivity and limiting the generalizability of results. Additionally, the diversity of cases might have been insufficient to capture the full range of real-world scenarios and challenges that ChatGPT could face. Furthermore, we did not thoroughly investigate ChatGPT's empathy and emotional intelligence in real-time, especially in acute situations. To address these limitations, future studies could expand the scope to include physician perspectives and simulated cases, develop a formal grading rubric or employ qualitative analysis for more structured and objective evaluations, increase sample size and case diversity to better assess ChatGPT's performance, and investigate its ability to provide empathic and emotionally intelligent responses in real-time, particularly in acute situations. By pursuing these future directions, we aim to enhance the understanding of ChatGPT's potential in healthcare and contribute to the development of more effective AI-assisted medical solutions.

## Conclusions

In summary, ChatGPT is a valuable resource for consultations regarding venomous snakebites, especially in remote or underserved areas with limited healthcare resources. Its real-time response capability, user-friendly language, and comprehensive advice make it an effective tool for general guidance and education. Additionally, its accessibility through various digital platforms and consistent delivery of information based on its training data enhance its value as an informative resource. However, it is crucial to acknowledge its limitations, such as outdated knowledge and the inability to personalize treatment for each case due to the unique characteristics of the region and the individual. Given the variability in the characteristics and severity of snakebites across different regions, it is essential to establish specific clinical guidelines for each region, which poses a challenge even for expert toxicologists. Despite this challenge, ChatGPT can offer generalized measures to manage snakebites. Moreover, its advantages, including its role in triage support and its educational function for snakebite prevention, identification, and management, underscore its significance as a supplementary source of information to complement professional medical consultation.
